# Mathematical model and genomics construction of developmental biology patterns using digital image technology

**DOI:** 10.3389/fgene.2022.956415

**Published:** 2022-08-10

**Authors:** Shiwei Ni, Fei Chen, Guolong Chen, Yufeng Yang

**Affiliations:** ^1^ Institute of Life Sciences, FuZhou University, FuZhou, Fujian, China; ^2^ School of Mathematics and Statistics, FuZhou University, FuZhou, Fujian, China

**Keywords:** digital image correlation, biology patterns, data-driven, medicine, microscopes

## Abstract

Biological pattern formation ensures that tissues and organs develop in the correct place and orientation within the body. A great deal has been learned about cell and tissue staining techniques, and today’s microscopes can capture digital images. A light microscope is an essential tool in biology and medicine. Analyzing the generated images will involve the creation of unique analytical techniques. Digital images of the material before and after deformation can be compared to assess how much strain and displacement the material responds. Furthermore, this article proposes Development Biology Patterns using Digital Image Technology (DBP-DIT) to cell image data in 2D, 3D, and time sequences. Engineered materials with high stiffness may now be characterized *via* digital image correlation. The proposed method of analyzing the mechanical characteristics of skin under various situations, such as one direction of stress and temperatures in the hundreds of degrees Celsius, is achievable using digital image correlation. A DBP-DIT approach to biological tissue modeling is based on digital image correlation (DIC) measurements to forecast the displacement field under unknown loading scenarios without presupposing a particular constitutive model form or owning knowledge of the material microstructure. A data-driven approach to modeling biological materials can be more successful than classical constitutive modeling if adequate data coverage and advice from partial physics constraints are available. The proposed procedures include a wide range of biological objectives, experimental designs, and laboratory preferences. The experimental results show that the proposed DBP-DIT achieves a high accuracy ratio of 99,3%, a sensitivity ratio of 98.7%, a specificity ratio of 98.6%, a probability index of 97.8%, a balanced classification ratio of 97.5%, and a low error rate of 38.6%.

## Summary of digital image technology

As digital imaging methods linked with light microscopy continue to develop exponentially, researchers in domains such as biology, medicine, and other sciences can generate vast image data in a wide range of exploration (Zhang et al., 2021). This potentially enormous amount of image data must be handled with care to enable the extraction of the necessary information in a timely and cost-effective manner (Emami et al., 2021). In this way, image analysis is not confined to analyzing the image that has been captured (Garreta et al., 2021). In many cases, it includes collaboration with people who gather pictures to decide on the best method to take while producing image data at the microscopes (An et al., 2021). It is usually preferable for image analysis and high-quality photos rather than attempting to make them suitable for later processing (Shao et al., 2021). It is critical to determine when it is appropriate to undertake a 2D study and when it is essential to expand the analysis to 3D (Jiao et al., 2021).

Images in 3D need more data and storage space. Still, they necessitate the development of new analysis techniques and more memory and processing capacity to manage the vast amounts of data that must be processed when the analysis is carried out (Blackiston et al., 2021). Over the last several years, professionals from biology and medicine and image analysis have collaborated to develop several significant research outcomes that have benefited both sides (Seyfferth et al., 2021; Tang et al., 2021). The study is considered an extension of the same undertaking—Digital Image Analysis of Cells—applications in 2D, 3D, and time. In recent years, great progress has been made in creating deep neural networks (NNs) to model heterogeneous materials (Nguyen et al., 2021).

These efforts include the neural operator learning approach, which aims to learn the mappings between dynamic system inputs and system states (Medialdea et al., 2021). The network may operate as a replacement for a solution operator in a dynamic system, and DBP-DIT is especially interesting (Heddleston et al., 2021). While neural operators have several advantages over classical neural networks, their most notable advantage is their generalizability to different input instances, which results in a computing advantage in prediction efficiency (Pourasad et al., 2021). A forward pass of the network is all that is required to solve for a new instance of the input parameter after the neural operator is trained (Granwehr and Hofer, 2021). When it comes to simulating the unknown physics rule of homogeneous materials, neural operators have shown to be quite effective (Sarkar et al., 2021).

DBP-DIT has explored the practicality of learning a material model for a latex material directly from digital image correlation (DIC) data (Fan et al., 2021). The suggested technique has shown that the learned solution operators substantially outperform the traditional constitutive model (Caleb et al., 2021). The primary goal is to research and enhance the current image analysis for new applications, including 3D applications where appropriate (Lürig et al., 2021). It has been explored if any new ways outperform the present ones. The image data utilized in this article depict cells from various investigations (Lewis et al., 2021). The images were taken utilizing various microscopy methods, both in 2D and in 3D, to get the desired results. In a time series of images, the passage of time adds a new depth to the images (Aljazaery et al., 2022).

The main contribution of this article is.

• The primary focus of this study, clinically and physiologically relevant traits, aims to establish a framework for and software tool for automatic identification and classification of microscopic biopsy pictures.
• An approach to biological tissue modeling based on data-driven workflow aims to predict the segmentation method based on digital image correlation (DIC) measurements under unseen loading scenarios or knowledge of the material microstructure.
• With its capacity to determine strain fields and translations down to the micron scale, DIC shows potential as a tool for examining other biological specimens in the laboratory.
• The mechanical reactions of a biological tissue specimen under various loading situations may be modeled using a neural operator learning approach.

The overall article structure follows: Section 1 explores the summary of digital image technology, Section 2 demonstrates the related works based on biology pattern recognition, Section 3 expresses the proposed methodology, and Section 4 depicts the results and discussion and finally concludes the article.

## Related works based on biology pattern recognition

As a potent method for examining cell states and activities at the single-cell level, single-cell RNA sequencing (scRNA-seq) has gained popularity in recent years (Li, 2021). As experimental platforms and bioinformatics methodologies have advanced rapidly over the last decade, scRNA-seq has become more affordable and viable for many medical facilities. Several cell and molecular pathways were involved in tissue formation, adult cell function, illness, and aging that had been studied using Drosophila as a model organism. Using scRNA-seq in Drosophila would address the obstacles and prospects of creating new findings.

Many characteristics, like number, distance, orientation, and location, were used to influence the functioning of protein networks in biological systems (Kong et al., 2021). It was possible to use DNA origami to create nanometer-precision scaffolding for protein assembly that could be controlled, programmed, and addressed. Several multidisciplinary studies recently realized the accurate building of DNA origami-based protein networks and the developing use in various fields. Some argue that DNA origami-based protein networks have been employed in various applications in the biomedical and enzymatic areas.

Hepatocellular carcinoma (HCC) was one of the world’s most deadly malignancies. Patient-specific medication screening for HCC was currently hampered by a shortage of reliable *in vitro* models (Xie et al., 2021). Three-dimensional bio-printed HCC (3DP-HCC) models taken from patient samples were effectively produced and developed well over lengthy periods. Using 3DP-HCC models, drug screening findings could be presented in an accessible and quantifiable manner. Finally, 3DP-HCC models were accurate *in vitro* models that had been dependable in long-term culture and could predict patient-specific medications for tailored therapy.

Digital image correlation (DIC) measures for assessing and improving additive manufacturing (AM) processes were reviewed in this study (Cunha et al., 2021). First, the DIC principle was revisited, and its application to various AM processes was discussed. An overview of the influence of *in situ* monitoring on AM processes was provided based on target themes such as defect characterization, residual stress assessment, geometric distortions, strain measurements, statistical model validation, and material characterization. An *in situ* measurement case study was provided for wire and arc additive manufacturing (WAAM), highlighting the prospects, problems, and solutions.

EA-LPME-SSHS-TAD was utilized to identify carbaryl in food samples using a digital image colorimetry approach (Jing et al., 2021). This method was used to extract 1-naphthol and separate it from the octanoic acid sample by altering the pH values of this solution. Tangerine compounds were created by combining the extracted solution MBDF connected to the TAD, which included 1-naphthol as one of its basic components.

Based on the above analysis, scRNA-seq, 3DP-HCC, DIC, and EA-LPME-SSHS-TAD, there are some issues such as low accuracy, sensitivity, and error rate. Therefore, this article proposed DBP-DIT digital image correlation (DIC) measurements and a data-driven approach to measuring biological materials.

## Development biology patterns using digital image technology (DBP-DIT)

Researchers may measure phenotypic changes in many cell populations using image-based cell profiling (Agboola, 2020). It provides the door for large-scale studies of biological systems by chemical and genetic manipulations. The typical approach for this technology is picture capture using high-throughput microscopy devices, followed by image processing methods. The suggested procedures demonstrate how a sequence of microscope images may be used to build high-quality image-based profiles. Worldwide laboratories are using image-based cell profiling to seek biological discoveries, and the tactics developed are based on their experience (Zhao et al., 2019; Chinnadurai and Sindhu, 2020). The proposed offered encompass choices that may fit varied biological aims, experimental designs, and laboratory preferences.

**FIGURE 1:**
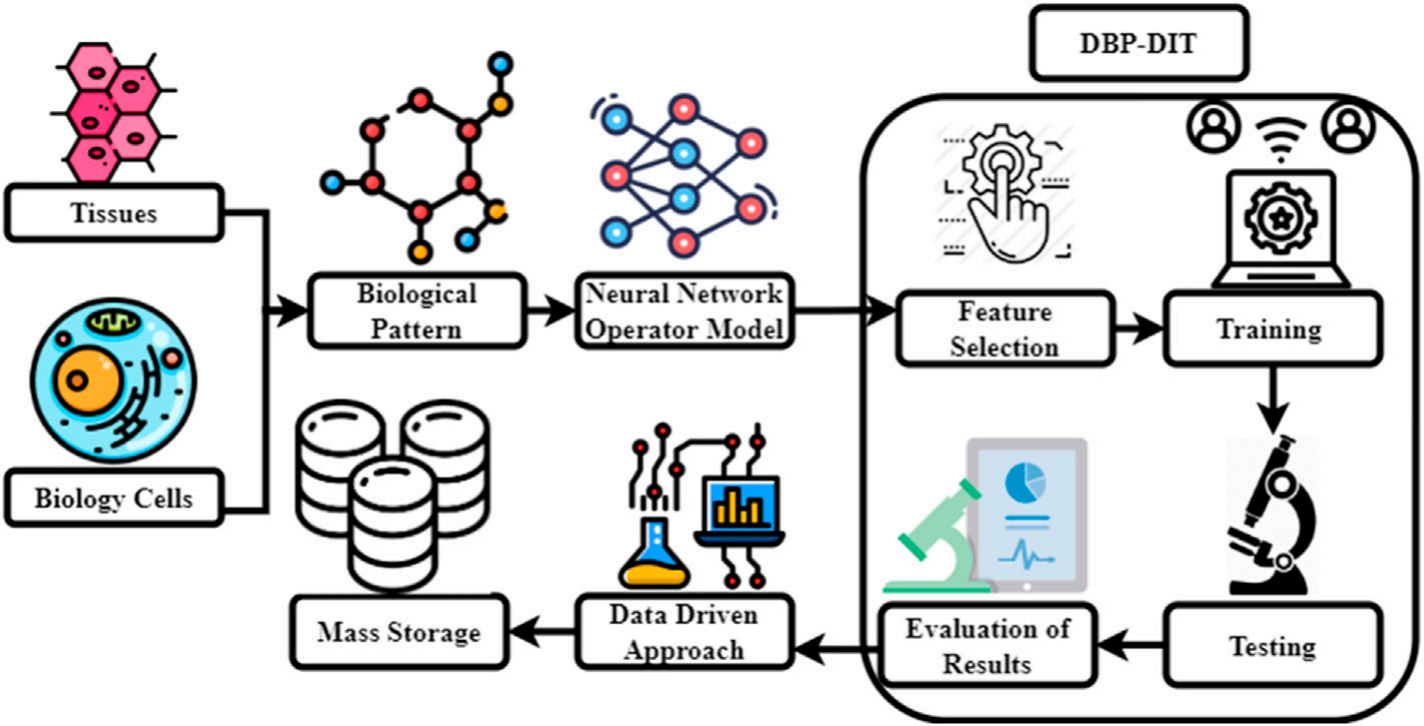
Proposed DBP-DIT.

Figure 1 expresses the proposed structure of DBP-DIT. In this diagram, there are five major functions such as *1*) tissues and biology cells, *2*) biological patterns, *3*) DBP-DIT, *4*) neural operator model and data-driven approach, and *5*) datastore.

i) Tissues and biology cells: tissue is a degree of biological order between cellular and a fully developed organ. A tissue is a group of lymphocytes and their matrix proteins from the same origin that work together to accomplish a specified task. Organs are then generated by the elements of the system together of many tissues. Multicellular creatures are organized into tissues, made up of physically and functionally identical cells and the intercellular material that connects them.
ii) Biological patterns: diverse processes lead to the emergence of biological patterns such as animal markings, animal segmentation, and phyllotaxis. “Pattern formation” refers to how cells in an embryo begin as homogeneous and gradually grow into various shapes and functions. There is the perfect coordination of genetic programming to generate complex tissues and organs. Numerous patterning genes have been discovered by genome sequencing and forward genetic screens, several of which are regulated in a tissue-specific way at certain stages of embryonic development.
iii) DBP-DIT: cell profiling using images is a high-throughput method for quantifying phenotypic variations across several cell types. Using chemical and genetic perturbations opens the door to investigating biological systems on a vast scale. Images are captured using high-throughput microscopy devices and then processed and analyzed using image processing software. A collection of high-quality microscope pictures may be used to construct high-quality image-based profiles. For assessing the mechanical characteristics of the skin, DIC seems to be a good approach. Because of its capacity to determine strain fields and translations down to the micron scale, DIC shows potential as a tool for analyzing other biological specimens. Research in biology, medicine, and industry may benefit from digital image correlation.
iv) Neural operator model and data-driven approach: using DIC-tracked displacement data rather than a pre-defined constitutive model or prior knowledge of tissue microstructure, this study aims to describe the mechanical response of biological tissue representations. Using digital image correlation (DIC), measurement techniques present a data-driven workflow for biological tissue modeling that attempts to predict the displacement field under unknown loading scenarios without postulating a specific constitutive model form or knowing anything about the material’s microstructure. DIC displacement tracking measurements of biaxial stretching protocols on the anterior leaflet of the valve are used to design a neural operator learning model for this purpose. Materials are treated as solution operators, with the material microstructure features learned implicitly and naturally integrated into network parameters, resulting in a material response model. We evaluate the framework’s predictability to that of a finite element model based on a phenomenology Fung-type model using different combinations of loading methods. By conducting distribution tests, we found that our method’s ability to anticipate the effects of diverse loading situations is superior to standard constitutive modeling by a factor of one to two.
v) Data store: there are a variety of storage and memory devices used in the vast majority of medical digital imaging systems. Each has a specific purpose dictated by its capabilities and constraints. While a picture is being captured, analyzed, and stored in RAM (random access memory).
A. Biological Pattern Structure Analysis

Heterocysts in the blue-green algae Anabaena are an example of a biological example of heterocysts, adding more peaks to the list of possibilities. For every 12–14 cells in the linear chain of cells, a normal cell becomes a bigger, non-dividing heterocyst (HetR) (black circles) that can no longer divide. In terms of distance from the existing heterocyst cell to be targeted for deletion. In the presence of HetR, heterocyst development is governed by the transcription factor HetR. HetR dimers trigger HetR transcription directly. Autocatalysis is predicted to be nonlinear, and dimerization demonstrates that this is true. Upon activation of HetR, the PatS (triangles) peptide is synthesized, which may cross intercellular junctions and attach to HetR. HetRDNA binding to PatS is no longer feasible if PatS is coupled and activator autocatalysis.

**FIGURE 2:**
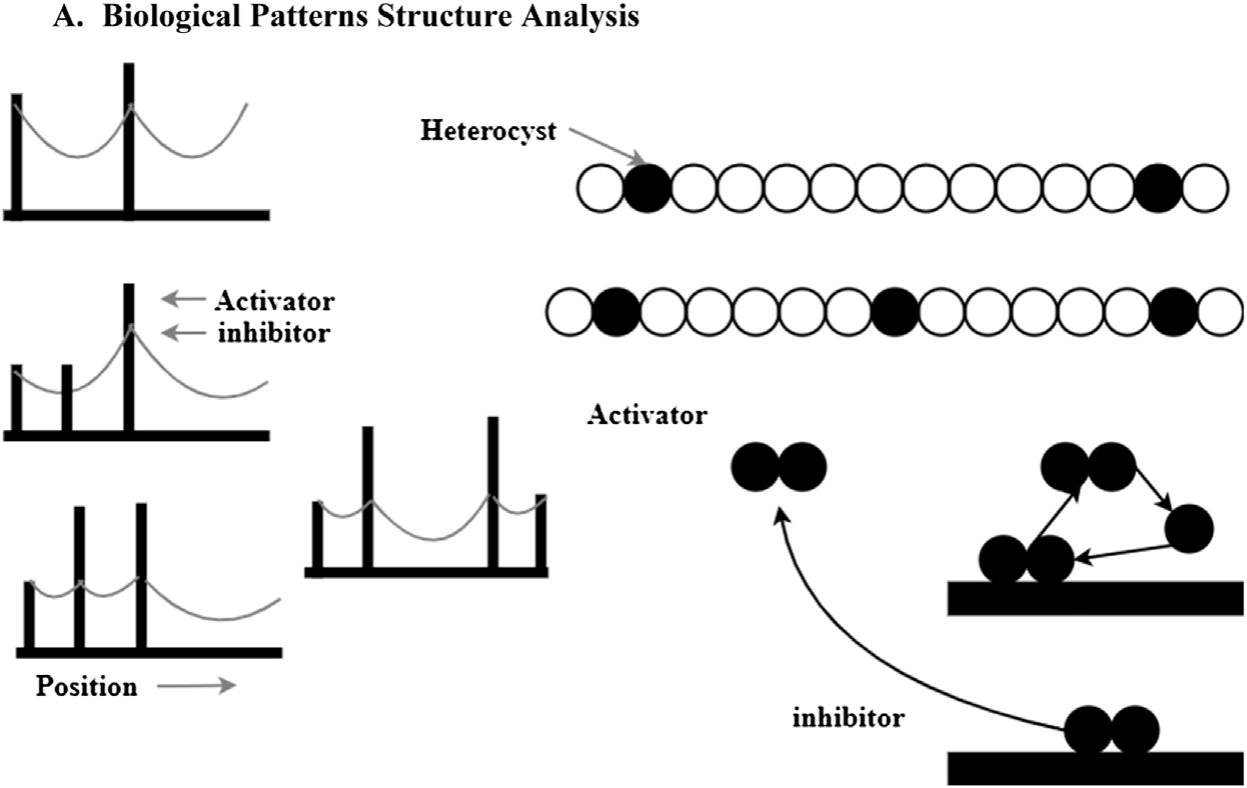
Biological pattern activator production.

Autocatalysis of the activator is initiated every time the inhibitor drops below the predetermined threshold level. More than one cell might activate simultaneously due to a low concentration of inhibitors. Due to competition, activation may occur in a single isolated cell. HetR mutations do not result in heterocyst formation, as predicted by our hypothesis. Most cells form heterocysts when PatS is mutated in contrast. Unlike the implantation of a new maximum in the absence of saturation, periodic patterns may form by splitting existing maxima in growing tissues shaped by systems with saturating activator production in Figure 2. The maxima of a plate broaden as it becomes saturated. The plateau-like activation pattern is size-regulated if the region into which the inhibitor canes cape grows. Due to the growing inhibitor level at the center of a maximum, activator synthesis at the center of a maximum may be lower than at the flanks from that point forward. To meet this criterion, an activator dimer must not be present in the active ingredient. The activator that causes heterocyst formation in Anabaena is dimerized.

**FIGURE 3:**
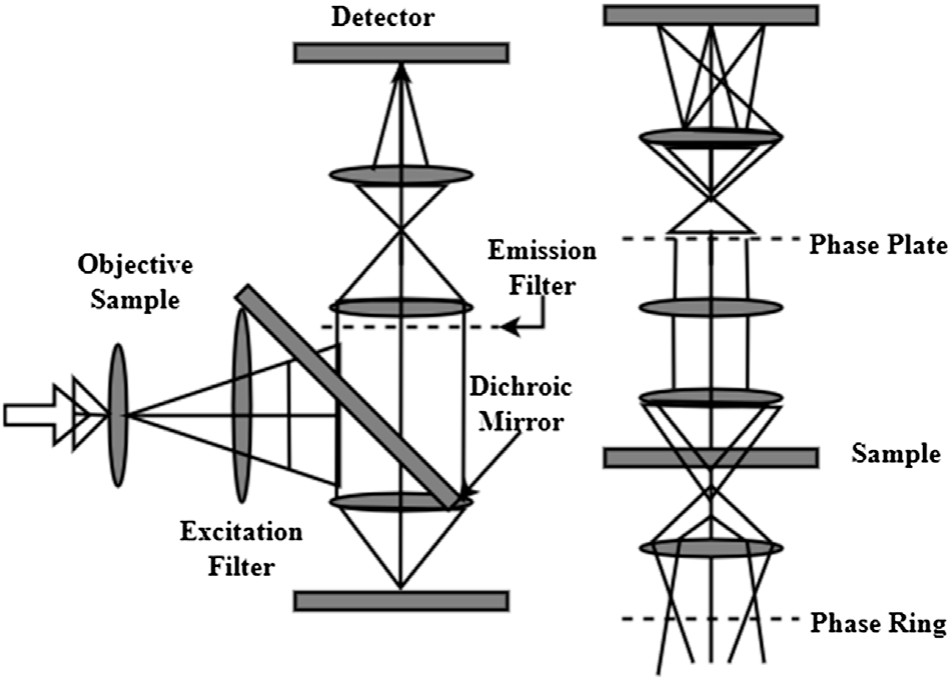
Microscopic-based bio-cell structure identification.

Contrasting amplitudes and wavelengths may be seen by the human eye and interpreted in terms of brightness and color by the eye. A bright-field microscope utilizes these two different contrasts to produce a picture of the material. With these sorts of microscopes, it is possible to see specimens that have some attribute that influences the quantity of light. It is possible to see an example of how light moves through a bright-field microscope in Figure 3. The specimen is illuminated by a light source (a) and a condenser (b). The light goes *via* an objective (c), a tube lens (d), and a projection lens (e) before it reaches the detector and is collected. It is the objective’s magnification that determines how huge the final projection. Staining specimens with a color may improve contrast, generally necessitating fixing the specimen, implying that the cells inside it are dead. When staining separate structures may choose from a variety of stains and all the colors will be recorded in the same picture. Eosin staining has been used in color spaces for cytology smears to identify cell nuclei. Finding cancerous samples is the study’s primary goal, and color may offer information about malignancy and other quantitative factors. Contrast may be enhanced in live-cell imaging using bright-field microscopy in various methods. When the samples are very low in absorption, utilizing additional approaches to enhance contrast is beneficial. Refraction rather than absorption is to blame for the apparent contrast in these situations. A digital image system is the greatest option for photographing several areas of a specimen in a time sequence using live cells. As a result, there will be less room for human mistakes when moving specimens between various places manually. For the cells to remain healthy for live-cell imaging requires a specifically regulated environment that resembles their natural habitat.

B) Digital Image Technology

**FIGURE 4:**
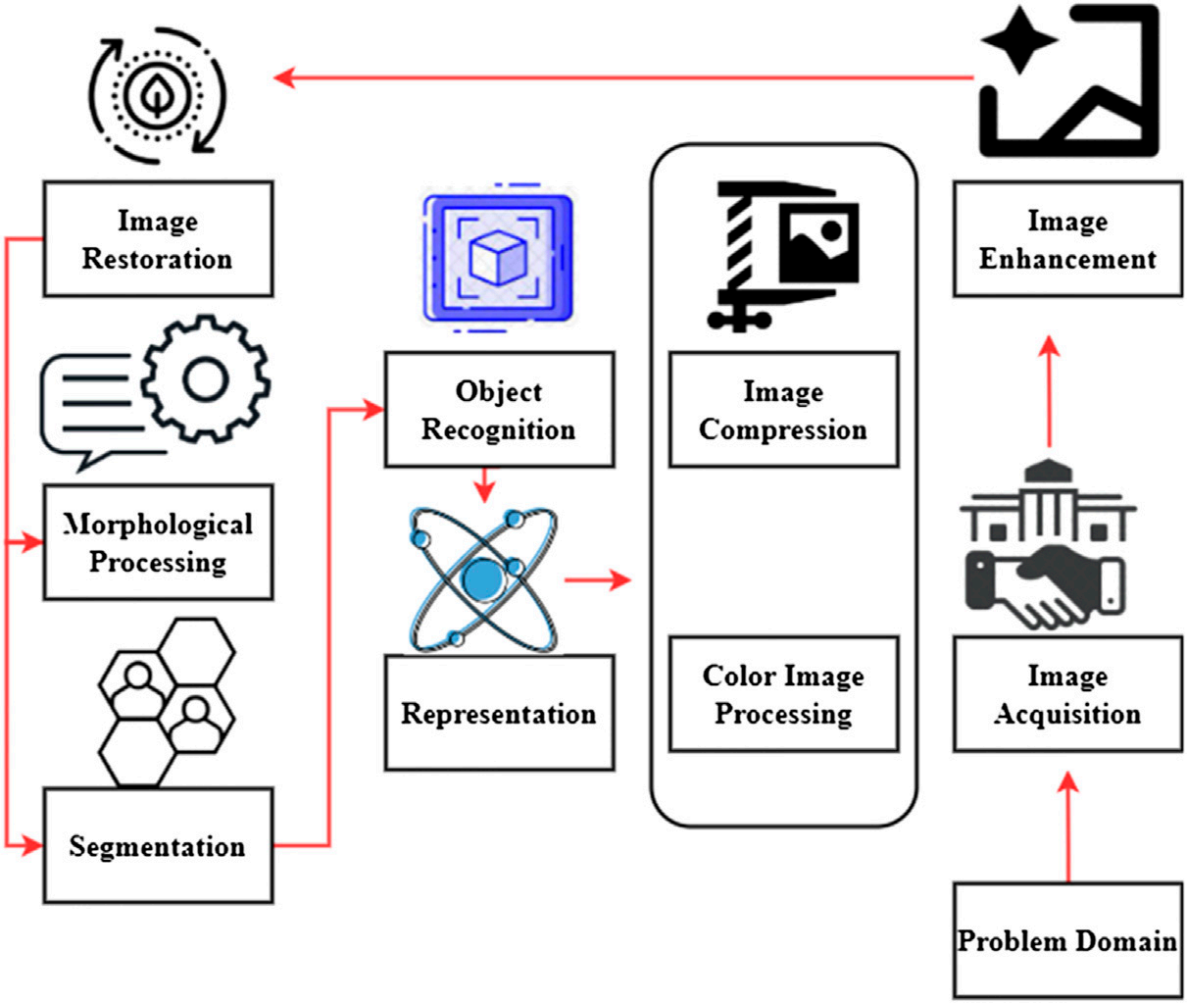
Basic functions of digital camera.

Figure 4 explores the basic functions of a digital camera. One may improve or extract information from a picture after converting it to digital form using image processing. An image, such as a video frame or a picture, may be used as an input for signal dispensing, and the output may be an image or the attributes associated with that image. In most cases, an image processing system treats pictures as two-dimensional signals and then applies pre-existing signal processing algorithms to those signals (Song and Brandt-Pearce, 2012).

First and foremost, digital image processing is a series of stages that begin with this one. If a photograph exists in digital form, acquiring it could not be easier. Scaling and other post-processing are frequent at the time of photo capture. In digital image processing, one of the easiest and most aesthetically appealing features is the ability to enhance one’s photographs.

To summarize, the goal of most image enhancement methods is to either reveal previously hidden details or draw attention to certain parts of a picture that the user finds interesting. Image restoration is concerned with enhancing the visual quality, and images may be restored using mathematical or probabilistic degeneration models rather than subjectively picture augmentation. Increasing usage of digital photographs *via* the Internet has made color image processing a growing subject of interest. In this context, digital color modeling and processing are included.

Images may be represented at a variety of resolutions using wavelets. Using data compression and the pyramidal representation of images, images are reduced to smaller and smaller sections. Compression methods reduce the storage or bandwidth needed to send a picture. Compression of data is very important for Internet-based applications. When it comes to techniques for extracting picture components that may be used to represent and describe a form of morphological processing. Procedures for image segmentation separate a picture into its component sections or objects. Automated picture segmentation is notoriously difficult in today’s world of digital image processing issues requiring the identification of individual objects; using a robust segmentation approach is an important first step toward a successful solution. Following a segmentation step, the raw pixel data may represent all components inside a region. The first step is to choose a representation to turn raw data into a form that computers can process. The description is the process of identifying characteristics that may be quantified or used to distinguish one item from another. Knowledge may be as easy as identifying areas on a picture where certain pieces of information are likely to be found, reducing the amount of time and effort needed to find them.

**FIGURE 5:**
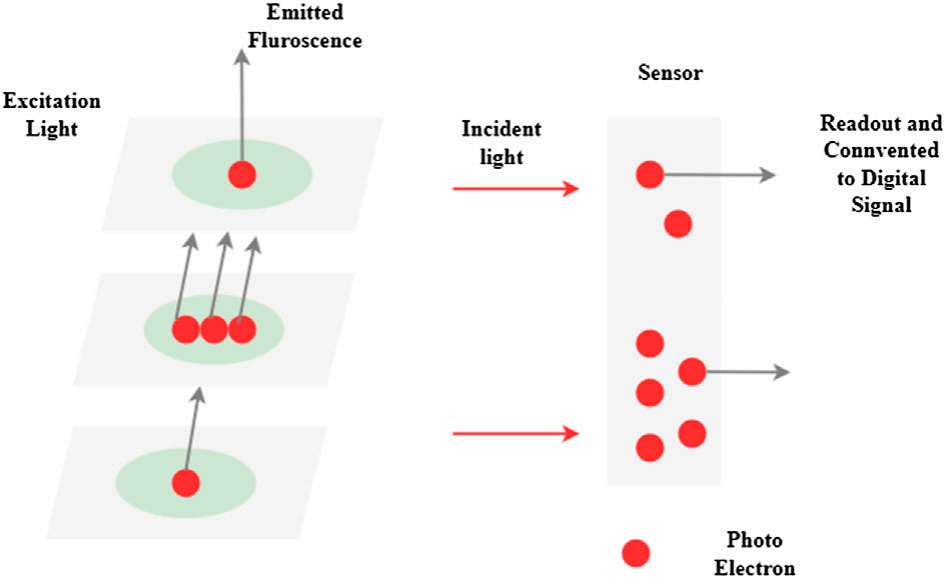
Digital camera-based biological signal analysis.

It examines the connection between biological signals and digital data from microscope cameras in this article shown in Figure 5. The best photos and data by understanding this connection and using it to advantage when designing an image collecting setup. Biological samples may be seen using a monochromatic camera in a microscope. Fluorescence dyes and proteins produce light recognized by the camera and converted into photoelectrons, subsequently detected as a digital signal by the microscope. Excitation light intensity, excitation and fluorescence emission/detection efficiency, and the number of tagged targets are all factors that affect the signal value. This includes the camera’s efficiency in converting light to digital signals. A signal is proportional to the quantity of target present when the same imaging equipment and image collection parameters are applied to various samples within a single experiment. A gene-edited sample may be quantitatively compared to its wild-type counterpart.

**FIGURE 6:**
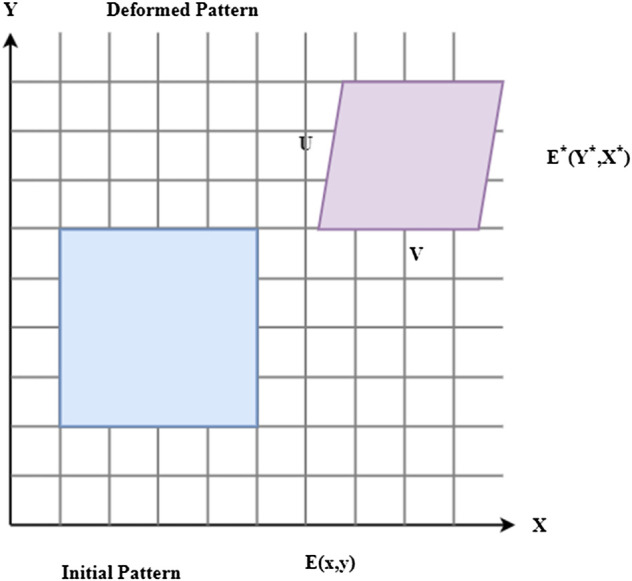
Digital image correlation.

Rigid engineering materials’ mechanical characteristics may now be accurately assessed *via* digital image correlation expressed in Figure 6. The strain and displacement response may be assessed by comparing digital material photographs before and after deformation. The present study applied this approach to soft biological materials, such as skin. This study shows that digital image correlation may be utilized to assess the mechanical characteristics of skin under a variety of situations, including displacement, uniaxial stress, and high temperature.

i) Connectivity of digital images

During the deformation process, $z^{*}$ represents the digital image that is created as a record of the discrete light intensities. Digital image correlation (DIC) $x^{*}$ implies that the speckle pattern Δv/Δx seen before deformation is connected to the speckle pattern imaged after deformation through rigid-body motion Δw/Δz and applied stresses are defined as

$$\begin{array}{c} z^{*}=z-v+\frac{\Delta v}{\Delta z}\partial z+\frac{\Delta v}{\Delta x}\partial x\\ x^{*}=x-w+\frac{\Delta w}{\Delta x}\partial x+\frac{\Delta w}{\Delta z}\partial z \end{array}\}$$ (1)

As presented in Eq. 1, z and x directional displacements are represented by v and w , respectively. Structural stresses in the horizontal $\frac{\Delta v}{\Delta z}$ and vertical axes $\frac{\Delta w}{\Delta x}$ are expressed as normal strains ∂z . Another factor ∂x that contributes to stress is shear.

Reduce the correlation coefficient to find such displacements and gradients of displacement. In most cases, the correlation coefficient is calculated using the least-squares B approach as follows:

$$B=\int {\left(g\left(z,x\right)+g^{*}\left(z^{*},x^{*}\right)\right)}^{2}dzdx$$ (2)

As presented in Eq. 2, where the pattern surface is denoted by g(z,x) . Digital image correlation enables speedy minimizing of this parameter $g^{*}\left(z^{*},x^{*}\right)$ , making it possible to estimate displacement and displacement gradients quickly.

C) Digital Image-based Biological Pattern Analysis

**FIGURE 7:**
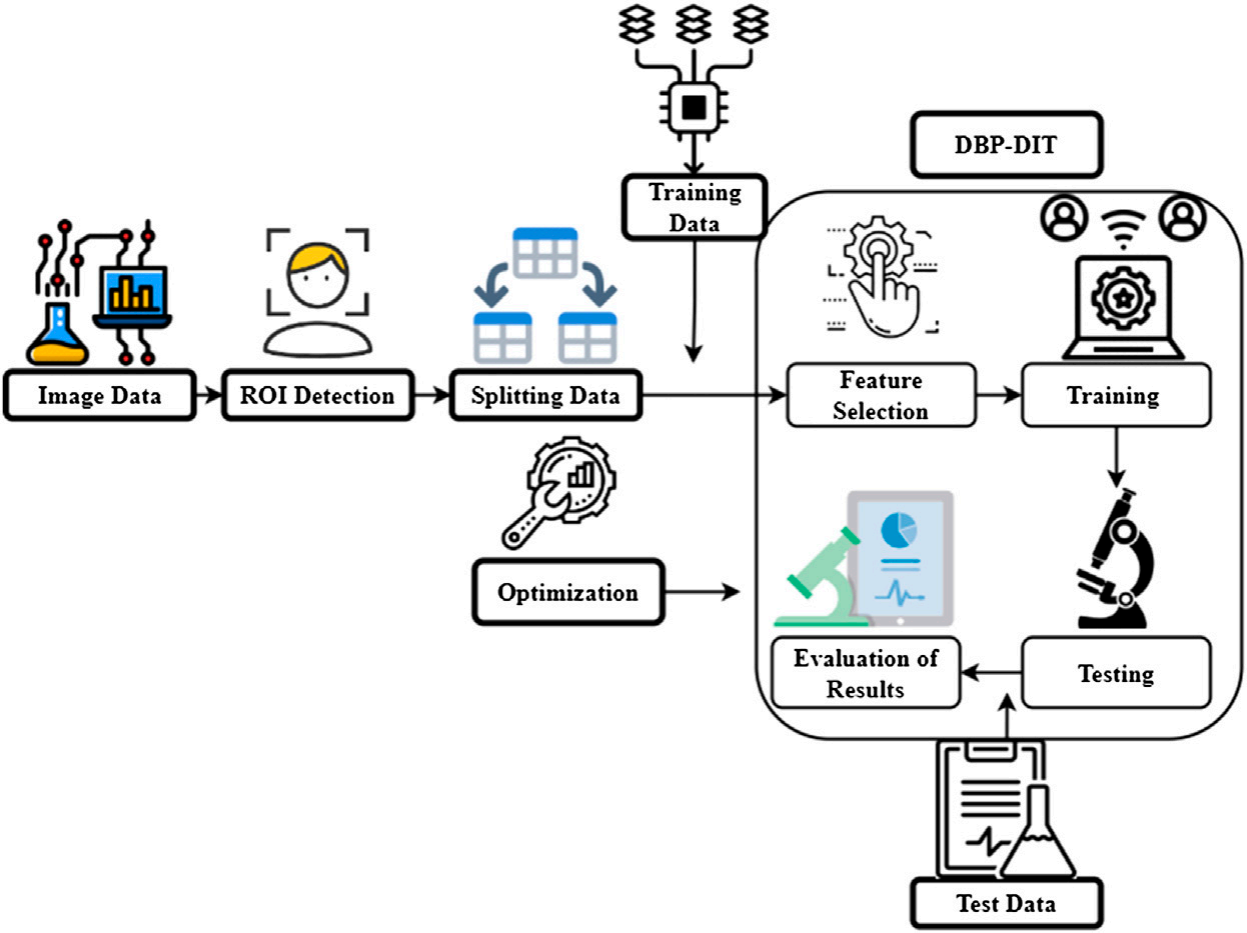
Digital image-based biological pattern recognition analysis.

Figure 7 determines the digital image-based biological pattern recognition analysis. Digital image-based biological pattern recognition (PR) aims to identify patterns in datasets and use them to identify new datasets. A subfield within artificial intelligence, PR is a kind of machine learning. There are two main categories of machine learning. Large picture collections have been generated by the automated image capturing systems that have been combined with laboratory automation. Pattern recognition is an efficient computing method for objectively analyzing image datasets. It is possible to teach a computer system to categorize unfamiliar objects based on the patterns discovered during the training process, known as supervised learning (PR). If there are no pre-defined classes, the computer system uses generic principles to partition or cluster the data. Protein localization may be identified automatically utilizing supervised learning techniques such as photos of probes placed in certain subcellular locations. Experiments using microarrays to analyze gene expression are a good illustration of unsupervised learning. Pattern recognition (PR) may benefit from numerous strategies to split pictures into ROIs, much as standard image processing systems focus on object identification, PR. To improve the response time or statistical significance, pixel resolution considerations, biasing PR algorithm to perform things of interest rather than the background, and centered or aligning objects with inherent orientation are three major reasons. Section finding regions of interest explains ROI identification strategies and tools in greater detail.

In the second step, the image content descriptors that describe the picture content quantitatively are extracted. Pixel intensity, edge, and color distribution may be represented in these statistics. Raw pixel dimensions may generally reach 1,000,000 a few dozen to a few hundred picture characteristics. A unique visual characteristic is represented by a feature value rather than a pixel value, representing the intensity of an X, Y location. The computing image aspects section provides a more in-depth explanation of the many typically used features. After that, the picture characteristics are used to derive inferences about the data. PR approaches often choose characteristics and apply weights depending on their ability to distinguish across classes. Classifier rules are then derived from the improved feature set. These two processes are part of the training phase of PR, where the aim is to identify the training pictures accurately. Excluded control photos are then used to evaluate the learned classifier. For the classifier to recognize new photos, it must be cross-validated to ensure that the images it has been trained with are recognized. A detailed description of feature selection and categorization may be found in this section. For a biological conclusion to be drawn, the researcher must analyze the findings of picture categorization in an experiment. In this interpretation, there are unique considerations for PR, which are explained more in the section on interpreting image classification output. Pre-defined classes reveal new linkages and establish new groups of feedback mechanisms and specified reward criteria for improving judgments in reward-based learning and semi-supervised learning. Supervised learning is used to analyze microscopic image files automatically in this instructional essay.

i) Processes of neural operator learning

The tissue microstructure and mechanical characteristics are unknown. Let ℋ be the unknown differential operator for the momentum balance v, and boundary conditions Ψ are as follows for a given boundary condition

$$\begin{array}{cc} ℋ\left[v\right]\left(z\right)=0, & z\in \Psi \\ v\left(z\right)=v_{E}\left(z\right), & z\in \Delta \Psi \; \end{array}\}$$ (3)

As presented in Eq. 3, an operator takes data as input v(z) and produces the displacement field as its output $v_{E}\left(z\right)$ , using neural networks (NNs) to integrate its descriptive power ΔΨ .

A sequence of observed function pairs ℱ using DIC measurements ${\left(v_{E}\right)}_{k}$ , where the input φ is a succession of boundary displacement loads z and the accompanying (possibly noisy) displacement area $v_{k}\left(z\right)$ is given as

$$\underset{\varphi \in \text{Ι}}{\max}\sum_{k=1}^{M}\left\|ℱ[{\left(v_{E}\right)}_{k};\varphi \right]\left(z\right)+v_{k}{\left(z\right)\|}^{2}N^{2}\left(\text{Ψ}\right)$$ (4)

As presented in Eq. 4, soft tissue response modeling $N^{2}\left(\text{Ψ}\right)$ is a challenge of learning the solution operator M of an undefined PDE system k using DIC data φ∈Ι .

ii) Implicit Fourier neural operators (IFNOs)

IFNOs are based on the notion of modeling the solution operator ${\overset{ˇ}{v}}_{E}\left(z\right)$ as a fixed point equation that readily matches the solution technique for displacement/damage fields ΔΨ in material modeling stated as

$${\overset{⌣}{v}}_{E}\left(z\right)=\{\begin{array}{cc} v_{E}\left(z\right), & if\;z\in \Delta \Psi \\ 0, & if\;z\in \Psi /\Delta \Psi \end{array}$$ (5)

As presented in Eq. 5, the microstructure $v_{E}\left(z\right)$ and characteristics of the material z are learned implicitly and gradually in the network parameters Ψ by learning the material responses directly from data.

Using the subscripts φ and z to denote the variables and operators associated with $v_{E}$ and $v_{k}\left(z\right)$ , the basic version of the neural operator learning structure ℱ is defined as

$${ℛ}_{data}\left(\varphi \right)=\sum_{k=1}^{M}\left\|ℱ[{\left(v_{E}\right)}_{k};\varphi \right]\left(z\right)+v_{k}{\left(z\right)\|}^{2}N^{2}\left(\text{Ψ}\right)$$ (6)

As presented in Eq. 6, analog ${ℛ}_{data}\left(\varphi \right)$ to segmented ordinary differential equations (ODEs) is the IFNOs’ iterative design k . Use the ideal network parameters acquired during the training of an IFNO of depth $N^{2}\left(\text{Ψ}\right)$ .

iii) Neural operators based on physics

Even though the neural operator model φ∈Ι depends on data $\varphi^{*}$ , its predictions cannot be the underlying physical laws ${ℱ}_{d}\left(\varphi \right)$ are defined as

$$\varphi^{*}=arg\underset{\varphi \in \text{Ι}}{\max}{ℱ}_{d}\left(\varphi \right)-\beta {ℱ}_{phy}\left(\varphi \right)$$ (7)

As presented in Eq. 7, enforce the underlying physical rules with soft penalty restrictions ${ℱ}_{phy}\left(\varphi \right)$ during model, training β to better exploit the neural operator learning methodology.

The no-permanent deformation hypothesis ${ℱ}_{phy}\left(\varphi \right)$ in an instance means that zero loading should lead to zero displacements ℱ[0;φ](z) for a specimen at rest.

$${ℱ}_{phy}\left(\varphi \right)=\left\|ℱ[0;\varphi \right]{\left(z\right)\|}^{2}N^{2}\left(\text{Ψ}\right)$$ (8)

As presented in Eq. 8, penalties to ensure that material under zero loading remains at zero deformation. As a consequence, it is expected that the physics-guided neural operator $N^{2}\left(\text{Ψ}\right)$ can enhance prediction accuracy in the low deformation domain.

**FIGURE 8:**
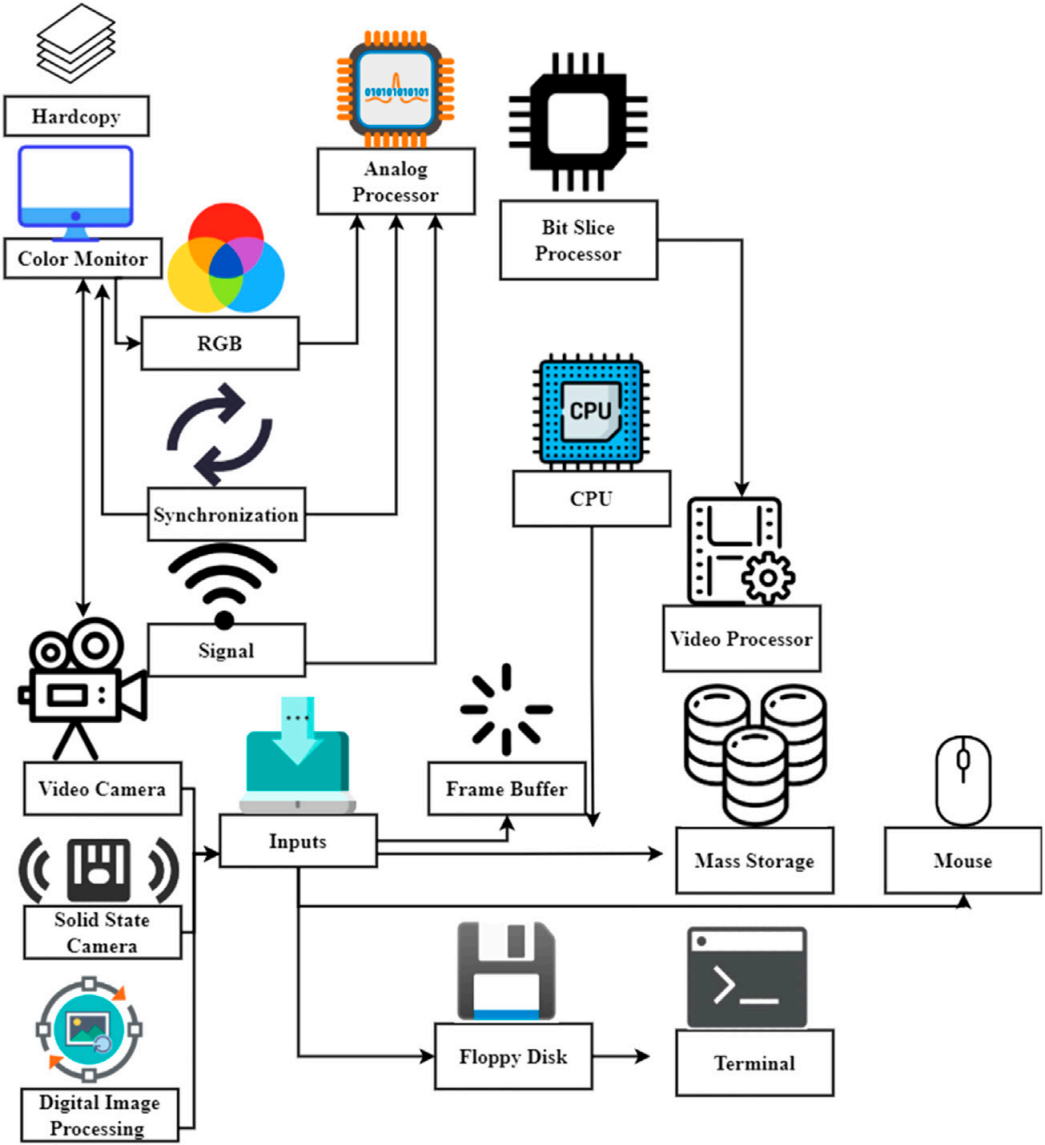
Digital image data acquisition and processing.

The system for acquiring and processing images in low light (GIPS) is illustrated in Figure 8. Either a TV source or a solid-state camera provides the input signals. Using an analog processor, images with a resolution of 512 by 512 pixels or fewer are captured from the inputs shown (upper). Input–output tables may apply real-time pixel alterations like contrast enhancement through quadratic scale expansion when the video signal intensity is high or low (LUT). A high-speed processor may conduct arithmetic operations before storing the data in a database. The microprocessor, the arithmetic logic unit, and a fast bit-slice processor are used to perform further image processing under the supervision of an integrated hardware–software system. The video recorder’s operations and the Q-bus memory map are controlled by other parts that are not shown. Semiconductor memory, frame buffers, and hard or soft disks may all be used to store images. The analog processor generates a raster display. One camera system now in use features a CCD area detector linked to an MCP amplifier circuit with a UV up to 5 × 10’ of adjustable electron intensity that a photocathode can gate.

High-resolution digitized signals are preamplified and routed *via* a high-speed ALU before being stored in memory that can be accessed through the microprocessor bus. Computer management of all camera operations is vital for protecting sensitive components (intensifiers) and ensuring the correct timing, synchronization, and gain for the desired results. The superscription of their names shows suppliers’ status of a system that can conduct comprehensive menu-driven picture acquisition and process parameters both in real-time and from saved data, the Q-bus components of the system and the requisite application for real-time photo editing and real-time image capture.

The difference between the target image F and the distorted source image $u_{j}$ , the soft landmark restrictions $F_{jnh}$ , and the *a priori* knowledge $u_{\rho }$ of the deformation field $F_{\rho }$ through the two independent measures that are linked to the divergence $F_{d}$ and curl gradients $F_{st}$ of the displacement sector are all considered as follows:

$$F=u_{j}F_{jnh}-u_{\rho }F_{\rho }+\left(u_{e}F_{d}-u_{t}F_{st}\right)$$ (9)

As shown in Eq. 9, when it comes to gradient-based optimizers $u_{t}$ , the multiresolution technique $u_{e}$ considerably enhances the resilience and performance of the algorithms.

i) Data Term

The objective of image registration $F_{jnh}$ is to identify a function that translates coordinates h(Z) from the destination image $J_{t}$ to the input; images are defined as

$$F_{jnh}=\int_{Z\in {\mathbb{Q}}^{2}}^{L}{\left(J_{r}\left(Z\right)+J_{t}\left(h\left(Z\right)\right)\right)}^{2}dzdx$$ (10)

As presented in Eq. 10, biological images $J_{r}\left(Z\right)$ are almost binary and are not suitable for histogram-based distance measurements $Z\in {\mathbb{Q}}^{2}$ .

The deformation field $F_{jnh}$ discovered can be different if the gray values φ in one of the images are modified since this measure of dissimilarity Z∈φ is sensitive to linear transformations $J_{r}\left(Z\right)$ of the image gray values are stated as

$$F_{jnh}=\frac{1}{\varphi }\sum_{Z\in \varphi }{\left(J_{r}\left(Z\right)+J_{t}\left(h\left(Z\right)\right)\right)}^{2}$$ (11)

As presented in Eq. 11, utilizing a normalizing process h(Z) , both images $J_{t}$ can be reduced to a single gray value framework using this dissimilarity measurement.

ii) Modeling of Deformity

A linear arrangement of B-splines h(Z) for the deformation field h(z,x) is as follows:

$$\begin{array}{l} h\left(Z\right)=h\left(z,x\right)\\ \;\;\;\;\;\;\;\;\;\;\;\;=\left(h_{1}\left(z,x\right),h_{2}\left(z,x\right)\right)\\ \;\;\;\;\;\;\;\;\;\;\;\;=\sum_{i,j\in X^{2}}\left(\begin{array}{c} d_{1},i,j\\ d_{2},i,j \end{array}\right)\alpha^{3}\left(\frac{z}{t_{z}}+i\right)\alpha^{3}\left(\frac{x}{t_{x}}+j\right) \end{array}$$ (12)

As presented in Eq. 12, using B-splines of degree three guarantees the continuation of the deformation’s second-order derivatives $d_{1}i,j$ . Spline approximations $d_{2}i,j$ , in particular, has a fourth-order of approximation $\alpha^{3}$ , which means that a spline approximation z , x of the genuine deformation X has a smaller inaccuracy $t_{x},t_{z}$ .

iii) Structures

As this landmark location $F_{\rho }$ can be affected by noise M , it has been decided to use soft limitations $\rho_{r}^{\left(m\right)}$ rather than accurate ones are defined as

$$F_{\rho }=\frac{1}{M}\sum_{m=1}^{M}{\|\rho }_{r}^{\left(m\right)}+h{\left(\rho_{t}^{\left(m\right)}\right)\|}^{2}$$ (13)

As presented in Eq. 13, column indices $\rho_{t}^{\left(m\right)}$ with all of the objective landmark’s components h , and is a matrix with the B-spline values m from the deformation model’s evaluation at its source landmarks.

iv) Regularization

The minimization problem $F_{C^{2}}$ can benefit from the smoothness of the deformation field $C^{2}$ as a regularization term $h_{1}$ Particularly, when there is limited information available as follows:

$$F_{C^{2}}=\int {\left\|C^{2}h_{1}\right\|}^{2}dzdx-\int {\left\|C^{2}h_{2}\right\|}^{2}dzdx$$ (14)

As presented in Eq. 14, the total differential operator $h_{2}$ , which is the square of the second derivative concerning it, can be found. Stress in a stretched elastic material is a factor in using this regularization term.

To reduce this energy, thin-plate splines $F_{rgh}$ must be established and designed as follows:

$$F_{rgh}=u_{e}F_{d}-u_{t}F_{st}$$ (15)

As presented in Eq. 15, $u_{e}F_{d}$ denotes the distance of the unique feature of the curl, and $u_{t}F_{st}$ is the slope of the scalar function.

Roughness energy $Z_{r}$ is evaluated just in the area $\Delta^{p_{1}-p_{2}}h_{k}$ where the goal image $\Delta^{p_{3}-p_{4}}h_{l}$ is specified, and all integrals are found $\Delta z^{p_{1}}\Delta x^{p_{2}}$ to be around the type as follows:

$$\int_{Z_{r}}\frac{\Delta^{p_{1}-p_{2}}h_{k}}{\Delta z^{p_{1}}\Delta x^{p_{2}}}\frac{\Delta^{p_{3}-p_{4}}h_{l}}{\Delta z^{p_{3}}\Delta x^{p_{4}}}dzdx={ℛ}_{k}^{D}S_{p_{1},p_{2,}p_{3},p_{4}}{ℛ}_{l}$$ (16)

As presented in Eq. 16, where $\Delta z^{p_{3}}\Delta x^{p_{4}}$ is a matrix containing all of the products in proper order ${ℛ}_{k}^{D}$ , and where $S_{p_{1},p_{2,}p_{3},p_{4}}$ are vectors containing all of the B-spline coefficients associated with deformation components ${ℛ}_{l}$ .

Integrals can be precalculated using closed formulas since B-splines are piecewise polynomials $D_{1}^{S}Q_{11}D_{1}$ . Consequently, three bilinear forms $D_{1}^{S}Q_{12}D_{2}$ can be used to calculate the roughness energy $F_{rgh}$ as follows:

$$F_{rgh}=D_{1}^{S}Q_{11}D_{1}-D_{1}^{S}Q_{12}D_{2}+D_{2}^{S}Q_{22}D_{2}$$ (17)

As presented in Eq. 17, the matrices can be precalculated, and the calculation is very quick and efficient. An additional benefit of this equation is that derivatives of the regularization term $D_{2}^{S}Q_{22}D_{2}$ can be easily computed.

The most difficult part of automatically detecting small biopsy pictures is classifying the images in the existing method. Classification may help determine whether a microscopic biopsy is benign. Section 3 concludes that our methodology has high accuracy, sensitivity, specificity, probability index, and less error rate to automatically overcome the difficulties of detecting small biopsy pictures.

## Result and discussion

Microscopy operations that create big picture databases are becoming more common thanks to automated image capture devices. These various datasets need powerful image analysis tools; there is a consensus that these systems do not exist. Most digital image analysis systems are designed to work with certain kinds of microscopy, contrast techniques, probes, and even cell types to acquire the best results in the studies they analyze. Since they were created for a certain subset of imaging modalities, this places considerable limitations on the kind of experiments that may be performed. Pattern recognition, which was initially designed for remote sensing, is increasingly being used in the biology area to address these restrictions. It educates the computer to recognize picture patterns rather than build algorithms or fine-tuning characteristics for particular image processing applications. This approach’s universality will allow data mining in large picture archives, leading to the frequent usage of objective and quantitative imaging tests. Pattern recognition and its use in biological and medical imaging video processing are briefly discussed here. Pattern recognition approaches for imaging tests are outlined and the practical considerations that may be employed to make the most efficient use of these techniques.

Dataset 1 description: a tumor is a mass of cells growing uncontrollably. A benign tumor is one in which the cells that make up the mass are unremarkable. They grew too much and formed a lump due to an error. Tumors are classified as malignant when they include cancerous cells, aberrant and capable of unchecked growth. This may have a major impact on prognosis and survival since prompt therapeutic care can be provided to patients. A more precise categorization of benign tumors might save patients from receiving therapies that are not essential. Each row has 30 independent features and one dependent feature. There are 114 rows in the test data, each with 30 distinct characteristics; https://www.kaggle.com/competitions/fcis-bio-2/overview.

Dataset 2 description: the competition’s goal is to help develop the best model feasible to link molecular information to a real biological reaction to the best of our ability with these data. The data have been provided with comma-separated values (CSV) for the ease of sharing. A row represents each molecule in this data collection. The chemical is seen to induce this reaction (1) in the first column or not. The second column comprises experimental data defining a hypothetical biological response (0). There are columns for molecular descriptors and estimated qualities that may capture some of the molecule’s features, such as its size, shape, or elemental composition. After normalizing the descriptor matrix, it is ready for use. The log loss measure estimates the likelihood that a chemical will cause a reaction. A sample that elicits a reaction is more likely to have evoked one if there are more samples; otherwise, it’s more likely that there was no response; https://www.kaggle.com/c/bioresponse.

Dataset 3 description: identifying and categorizing blood samples from patients are often a step in diagnosing disorders with a blood component. Detecting and classifying blood cell kinds using automated approaches have significant medicinal significance.

There are 12,500 more images of blood cells (in JPEG format) in this collection, along with cell-type designations (CSV). There are over 3,000 images in all, divided across four distinct files, one for each of the four categories of cells (according to cell type). Lymphocytes, monocytes, and neutrophils are the four cell types. Originally 410 pictures (pre-augmentation), as well as two extra subtype labels (WBC versus WBC) and also bounding boxes for each cell in each of these 410 photos (JPEG + XML information), are included in the collection. Additionally, the “dataset-masters” folders each include 410 photos of blood cells (JPEG + XML), whereas “dataset2-masters” has 2,500 enhanced images (JPEG + CSV) and four extra subtype labels (JPEG + CSV). Dataset-master contains just eighty-eight (88), thirty-three (33) (33), and twenty-one (21) (207) (3,000) enhanced photos for each of the four classes. Identifying and categorizing blood samples from patients are often a step in diagnosing disorders with a blood component. Detecting and classifying blood cell kinds using automated approaches has significant medicinal significance; https://www.kaggle.com/datasets/paultimothymooney/blood-cells.

Dataset 4 description: many individuals have to go without certain medications and medical procedures because of the rising costs. There is a categorization project in need of your assistance.

The time it takes to bring innovative medicines to market is one of the most unexpected reasons for the high cost. Although technology and science have advanced, the pace of research and development has not kept up. On average, it takes more than 10 years and hundreds of millions of dollars to develop new medicines. Artificial intelligence can revolutionize and speed up the drug development process, according to the producers of the industry’s biggest archive of biological pictures, all developed in-house. Efforts in this area might help researchers better understand how medications interact with human cells. Distinguishing experimental noise from biological signals is the main goal of this competition. Images of cells will be assigned to one of 1,108 distinct genetic mutations. They help reduce the noise caused by technical execution and environmental variance in subsequent tests. This might significantly impact the industry’s capacity to represent cellular pictures by the relevant biology.

On the other hand, artificial intelligence can significantly reduce treatment costs while ensuring that these therapies reach patients more quickly. The NeurIPS 2019 competition track includes this competition, and there will be an opportunity for the winners to present their ideas during a workshop.

For 51 experiments, identical siRNAs (essentially genetic perturbations) have been administered to numerous cell lines. There are four plates in each batch, with 308 filled wells. A total of six different imaging channels and two different locations were used to capture microscopical pictures of each well. Every well may not be filled or every siRNA present in every batch; https://www.kaggle.com/c/recursion-cellular-image-classification.

In this article, in Section 4 result and discussion analysis, *x*-axis takes several image data, and the *y*-axis takes the performance of classifiers is evaluated using a 2 × 2 matrix of confusion and the values of true positive (TP), true negative (TN), and false positive (FP). False-negative (FN) was determined. Accuracy, sensitivity, and specificity were determined using the methods above.

i) Accuracy Ratio (%)

Transforming neurotrophic beta ligands promote downstream gene transcription in the nucleus *via* activating intracellular SMADs. Gene expression is controlled by the two receptor-regulated SMADs and one coSMAD that forms a trimer in the body. Biological findings imply that the Smad complexes and the individual Smad proteins have variable kinetics and that the Smad protein activations are time-regulated. Because of this, the complexes had to be located concerning the cell’s outermost layer. The focus here is on accurately determining the boundaries of cell nuclei in digital images. Point-like signals must be accurately identified to infer biological implications from the data. The number of properly identified samples determines the accuracy of a classification method

$$Accuracy=\frac{TP+TN}{M}\times 100$$ (18)

**FIGURE 9:**
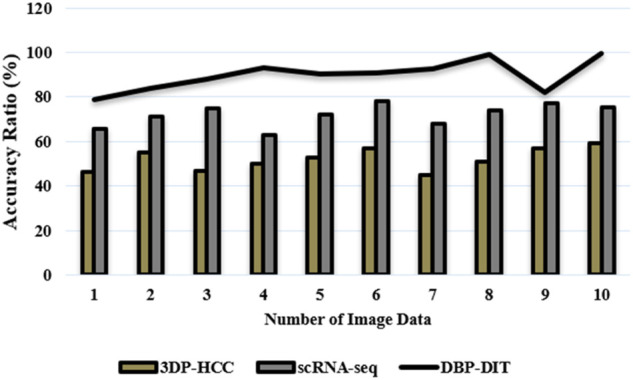
Accuracy ratio (%).

Figure 9 and Eq. 18 have discussed the accuracy ratio of digital images using datasets (Agboola, 2020), (Chinnadurai and Sindhu, 2020), where the number of samples in the microscopic biopsy images is aM . Patients who have been correctly diagnosed will have their disease surgically removed by matching histology samples, which will be included with the first smears. To build a database of validated samples that fresh samples may be quickly and accurately identified in the medium to long term.

ii) Sensitivity Ratio (%)

**FIGURE 10:**
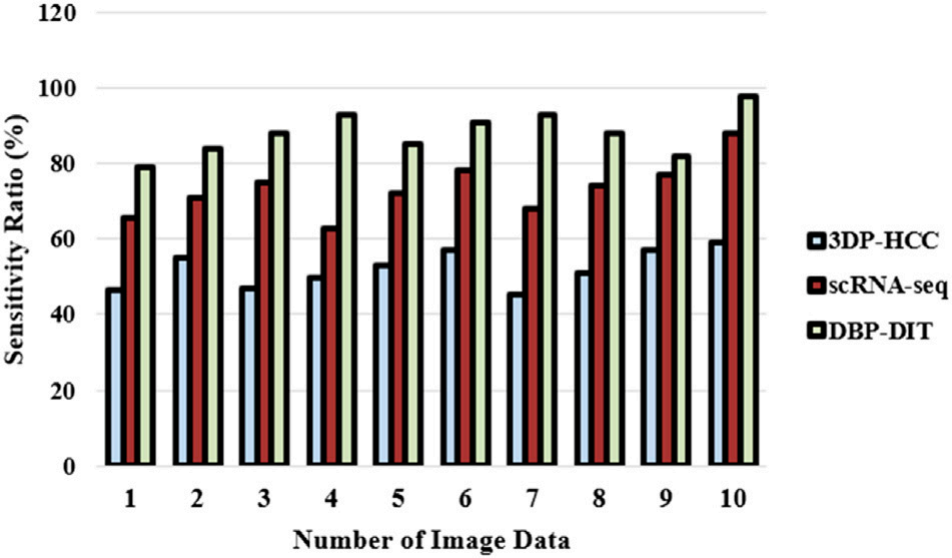
Sensitivity ratio (%).

Point-like signal identification in the digital image technology has been shown and evaluated for its resistance to noise, resolution power, and signal strength sensitivity compared to other regularly used approaches. The method’s performance on simulated data is promising, and the findings are much better than those of previous techniques. The method’s capacity to be applied in digital image technology for signal recognition and localization is further shown by experiments conducted on actual picture data from mitotic research. Figure 10 explores the sensitivity ratio

$$Sensitivity=\frac{TP}{Tp+FN}$$ (19)

Figure 10 and Eq. 19 express the sensitivity ratio based on the dataset (Chinnadurai and Sindhu, 2020), (Zhao et al., 2019). It is possible to see a certain macromolecule or cell component even when there is a large abundance of other species; low concentrations may be quantified because of the intrinsic sensitivity of emission rather than absorption processes.

iii) Specificity Ratio (%)

**FIGURE 11:**
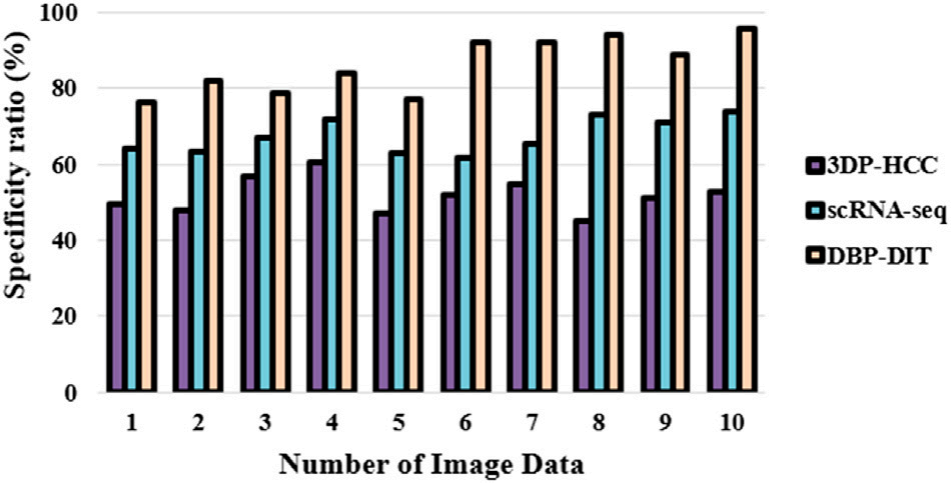
Specificity ratio (%).

A digital image technology may be conceived of as a 3D data set when watching living cells in time-lapse images, which are required to study the development of the cells. Once the tracking process is complete, it must deal with cells that split, merge, and form clusters over time. Figure 11 shows the specificity ratio

$$Specificty=\frac{TN}{TN+FP}$$ (20)

It is now possible to pinpoint particular protein complexes within cells using fluorescence microscopy with great specificity to the digital image technology through datasets (Song and Brandt-Pearce, 2012). A proper analysis can be measured to a certain extent if the antibody and the detection technique are highly specific. Low-light signals from biological samples can now be quantified using integrated systems that include microscopes, sensors, and image-processing software. Phosphorus-conjugated for antibodies and other ligands or enzyme substrates serve as specificity providers.

iv) Balanced Classification Rate (%)

**FIGURE 12:**
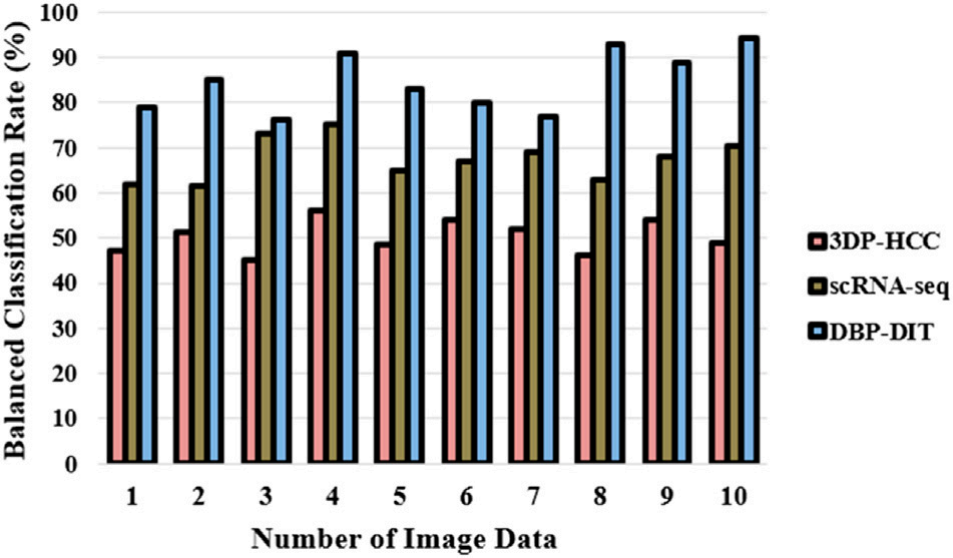
Balanced classification rate (%).

A classification model’s performance may be evaluated using the measure of balanced accuracy. To get a good mix of sensitivity and specificity, use the geometric mean of these two metrics. It is shown in the form of Eq. 21 and Figure 12​

$$Balanced\;Classification\;Rate\left(BCR\right)=\sqrt{Specificity\times Sensitivity}$$ (21)

As described in Eq. 21, the balanced classification rate has been expressed by the utilization of the dataset (Chinnadurai and Sindhu, 2020), (Song and Brandt-Pearce, 2012). It is possible to measure a binary classifier’s accuracy using a balanced classification rate metric. If one of the two classes occurs much more often than the other is extremely valuable. Anomaly identification and the existence of disease are two examples where this occurs often. It is best to utilize the measure of balanced accuracy with unbalanced data. As a result, it does not mislead by presenting too skewed data in one direction or the other.

v) Probability Index (%)

**FIGURE 13:**
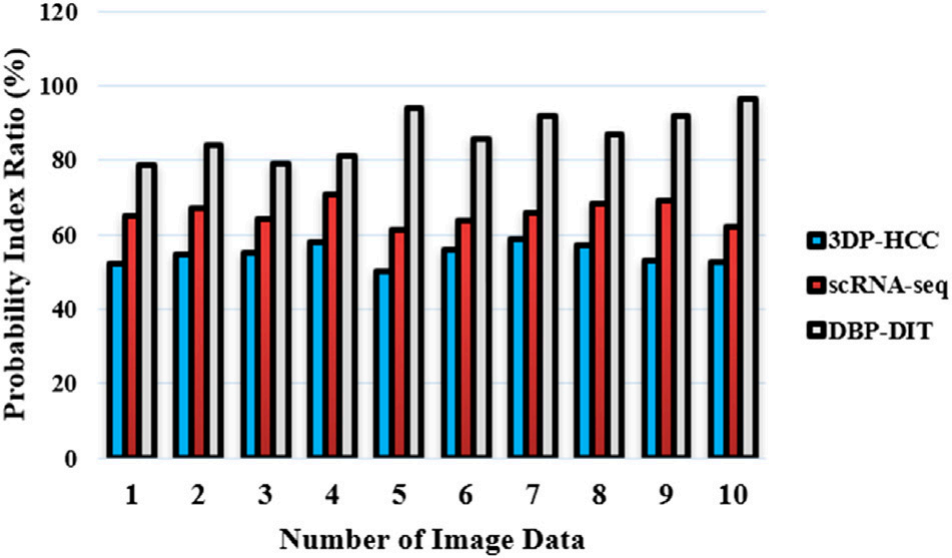
Probability index ratio (%).

A random probability index is a novel approach developed to segment the section of an image, which is most crucial for determining the true complexities involved in the portion of a body. Segmentation algorithms’ quality may be gauged non-parametrically using a random probability index. Where S is a random index test and ground truth H , and $H_{l}$ represents the ground truth segmentation. Figure 13 achieves the probability index

$$PRI\left(T_{test},H_{l}\right)=\frac{1}{\left(M/2\right)}\sum_{\forall j,i\&j<i}\left[D_{ji}Q_{ji}+\left(1-D_{ji}\right)\left(1-Q_{ji}\right)\right]$$ (22)

As found in Eq. 22, the probability index has been demonstrated through datasets (Zhao et al., 2019), (Song and Brandt-Pearce, 2012). The amount of adjacent pixels with much the same label and pixel pairings with various labels in both images is added to arrive at this result t and H and then dividing it by the total number of pixel pairs. The PRI is calculated based on a collection of ground truth segmentations $H_{l}$ such that $D_{ji}$ is a pixel pair that is described in this occurrence (j,i) in the test image $T_{test}$ that has the same or different label.

vi) Error Rate (%)

**FIGURE 14:**
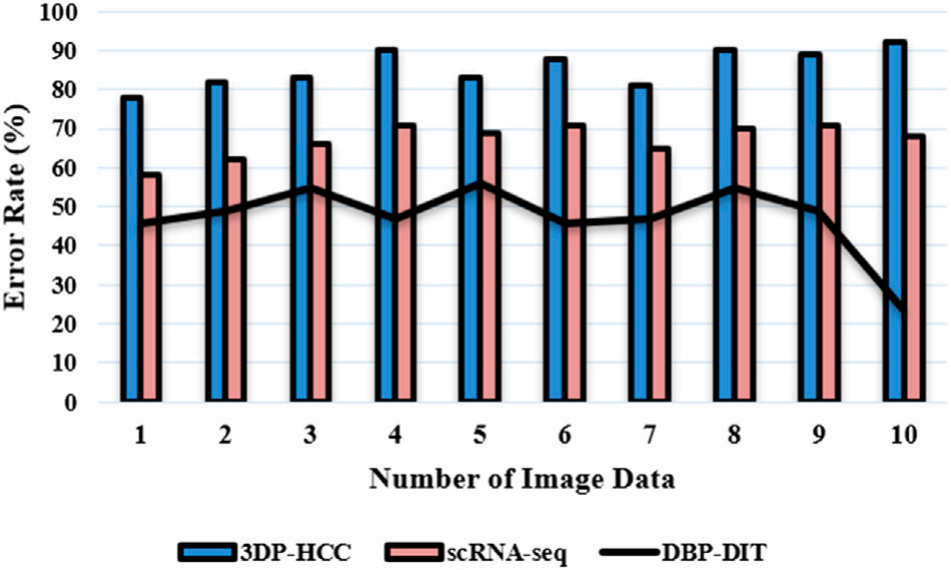
Error rate (%).

Estimates of the error range from 10 to 100%. $Q_{l}$ says a pixel is included in segments $T_{j}$ and $h_{j}$ such that t∈T,H∈h , where t indicates the set of segments creating a segmentation method that is used to be assessed and H indicates the collection of reference points. This is an example of a segmentation problem. Figure 14 deliberates the error rate

$$F\left(T_{i},H_{i},q_{l}\right)=\frac{\left|O\left(T_{i},q_{l}\right)\backslash O\left(H_{i},q_{l}\right)\right|}{\left|O\left(T_{i},q_{l}\right)\right|}$$ (23)

A measure of error is first calculated using (23) to compute the errors, and m indicates the collection of transformation operations and O(y,x) denotes the fixed pixels belonging to axis y that contains x in the axis. An image’s error rate may be calculated by multiplying the image’s total number of pixels by four using a dataset (Zhao et al., 2019), (Song and Brandt-Pearce, 2012). Segmentation errors may be quantified using error.

The proposed method achieves high outcomes when compared to scRNA-seq (Li, 2021), 3DP-HCC (Xie et al., 2021), DIC (Cunha et al., 2021), EA-LPME-SSHS-TAD (Jing et al., 2021) through dataset (Agboola, 2020), (Chinnadurai and Sindhu, 2020), (Zhao et al., 2019), (Song and Brandt-Pearce, 2012).

## Conclusion

This article enhances the microscopic biopsy image data augmentation applied and is used for laboratory information about connective, epithelial, muscle, and nervous tissues. Our approach incorporates and improves upon a number of the most effective medical imaging modalities. Automated identification and categorization of microscopic biopsy images are detailed based on clinically relevant and physiologically interpretable properties: tissue-level microscopic findings guide cell and nucleus categorization. Further, it has been shown that the suggested approach performs better in the connective tissue-type sample test instances than in other test cases. Analyzing the mechanical characteristics of skin under various situations, such as one direction of stress and temperature in the thousands of degrees celsius may be done *via* digital image correlation. Modeling biological tissues using digital image correlation (DIC) data, without a specific constitutive model or knowledge of the material microstructure, predicts the transformation function under unknown loading situations. Simulating the mechanical response of real tissue specimens under diverse stress situations using a neural operator learning approach. The experimental results show that the proposed DBP-DIT achieves a high accuracy ratio of 99.3%, a sensitivity ratio of 98.7%, a specificity ratio of 98.6%, a probability index of 97.8%,a balanced classification ratio of 97.5%, and a low error rate of 38.6%.

## Data Availability

The original contributions presented in the study are included in the article/Supplementary Material; further inquiries can be directed to the corresponding author.
